# Supplementary material for: Probing the rules of cell coordination in live tissues by interpretable machine learning based on graph neural networks
Source: PLoS Comput Biol. 2022 Sep 6;18(9):e1010477. doi: 10.1371/journal.pcbi.1010477 (PMC9481156; doi:10.1371/journal.pcbi.1010477)
Supplement: S1 Appendix — Fig A: Dependence of the number of iteration of the spatial MP on the performance. We apply the bidirectional spatiotemporal GNN model with sum or mean aggregation for the hind paw data under various feature conditions. (i) Schematic of the interaction range achieved by the different numbers of iterations of the spatial MP. (ii) The AUC for each cell fate label obtained by averaging over six trained models. Error bar: standard deviation. Fig B: Learning curves and confusion matrix. The cell external model with mean aggregation was applied for the hind paw data with the feature condition: (Area, G1 signal, NFB, Random) = (−, −, +, +). The curves of (i) the weighted softmax-cross-entropy loss, (ii) macro-F1 score, (iii) recall and (iv) precision for training and test data, respectively. The vertical dashed lines indicate the epoch at which the model achieves the best macro-F1 score. We evaluated the models with the best macro-F1 score in the main text. As shown in (i), the test loss decreases only slightly due to the low predictability in this setting; higher AUC can be achieved even for high loss within this close-to-random regime. In (v) and (vi), the confusion matrices of the training and test data averaged over 6 models which achieve the best macro-F1 scores are shown. The standard deviation is also shown. Note that the total number of events are fewer than the value reported in Fig L since cells near the spatio-temporal boundaries of the data set were not used. Fig C: Statistical properties of the hind paw data. (i,v) Histogram of relative frequency and the boxplot of the normalized area. (ii,vi,vii) Histogram of relative frequency, the boxplot, and the plot of the average of the normalized G1 signal. The error bar in (vii) is the standard deviation. (iii) The correlation matrix between the number of neighboring cells and normalized area. (iv,viii) Histogram of relative frequency, the boxplot of the number of neighboring cells. In (v,vi,viii), the significance ob [file pcbi.1010477.s001.pdf]

SUPPLEMENTAL INFORMATION
Probing the rules of cell coordination in live tissues by interpretable machine learning based on graph neural networks

Takaki Yamamoto,[1,*] Katie Cockburn,[2,3] Valentina Greco,[2,4] and Kyogo Kawaguchi[1,5,6,†]

[1]*Nonequilibrium Physics of Living Matter RIKEN Hakubi Research Team, RIKEN Center for Biosystems Dynamics Research, Kobe, Japan*
[2]*Department of Genetics, Yale School of Medicine, New Haven, Connecticut, United States of America*
[3]*Department of Biochemistry and Rosalind & Morris Goodman Cancer Institute, McGill University, Montreal, Quebec, Canada*
[4]*Departments of Cell Biology and Dermatology, Yale Stem Cell Center, Yale Cancer Center, Yale School of Medicine, New Haven, Connecticut, United States of America*
[5]*RIKEN Cluster for Pioneering Research, Kobe, Japan*
[6]*Universal Biology Institute, The University of Tokyo, Tokyo, Japan*

[*] yamamototakaki1212@gmail.com
[†] kyogo.kawaguchi@riken.jp

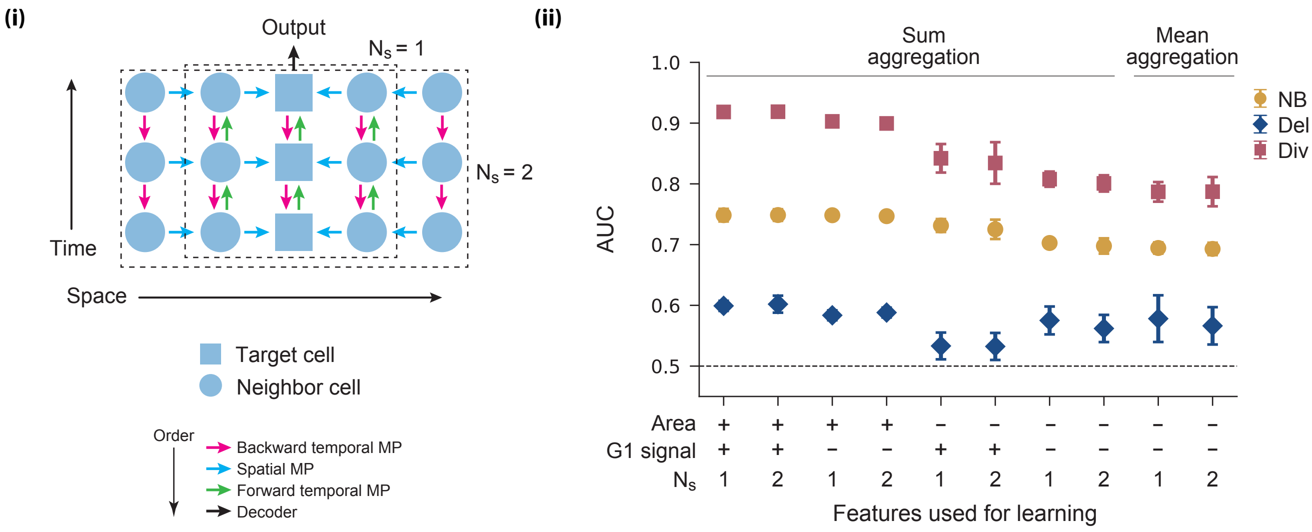

Fig A. **Dependence of the number of iteration of the spatial MP on the performance.** We apply the bidirectional spatiotemporal GNN model with sum or mean aggregation for the hind paw data under various feature conditions. (i) Schematic of the interaction range achieved by the different numbers of iterations of the spatial MP. (ii) The AUC for each cell fate label obtained by averaging over six trained models. Error bar: standard deviation.

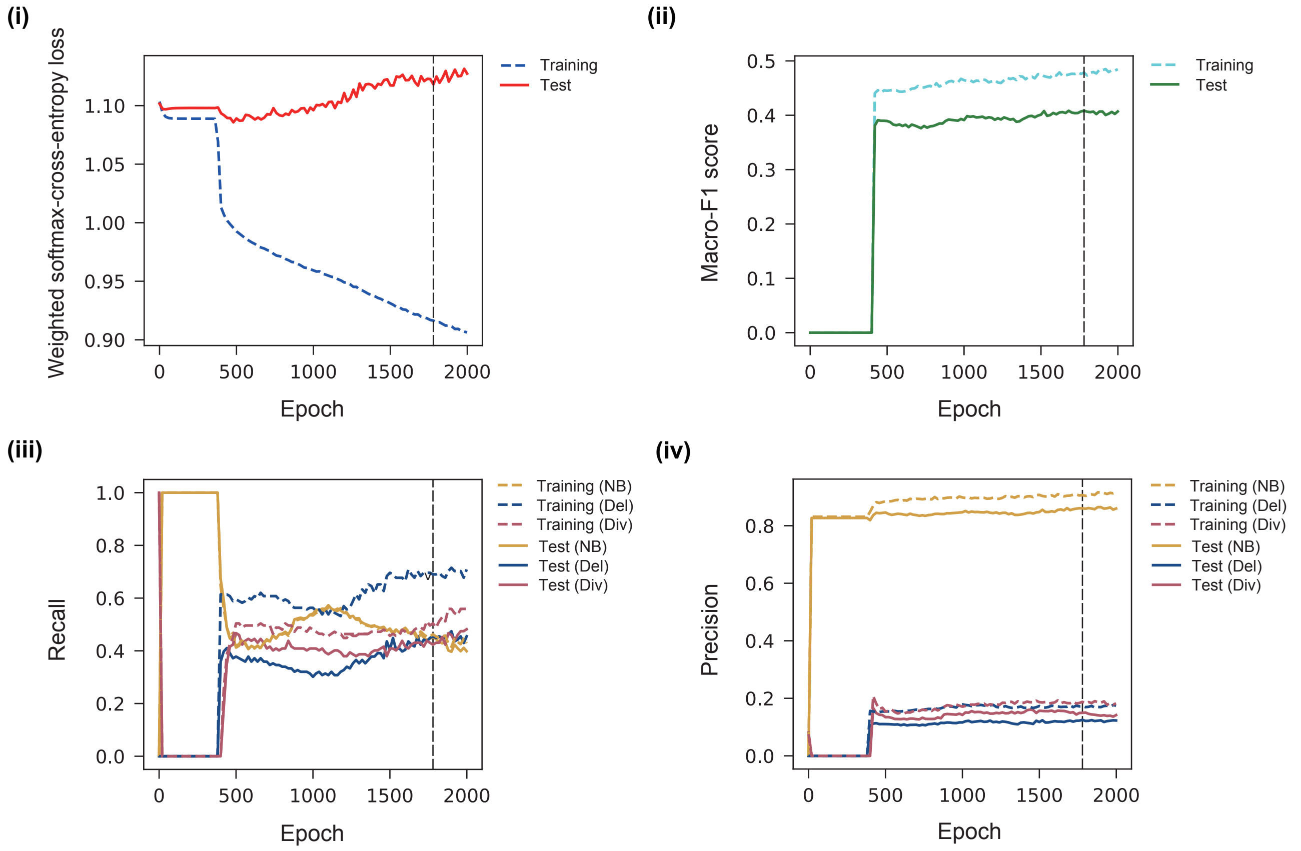

(v) Training

Predicted class (rows: True class)

	NB	Del	Div	Total
NB	615±69	551±47	238±72	1404
Del	25±11	112±7	22±8	158
Div	40±15	29±3	57±16	127

(vi) Test

Predicted class (rows: True class)

	NB	Del	Div	Total
NB	623±70	507±52	270±94	1400
Del	58±9	75±5	23±10	156
Div	46±10	37±4	54±13	137

Fig B. **Learning curves and confusion matrix.** The cell external model with mean aggregation was applied for the hind paw data with the feature condition: (Area, G1 signal, NFB, Random)=(−, −, +, +). The curves of (i) the weighted softmax-cross-entropy loss, (ii) macro-F1 score, (iii) recall and (iv) precision for training and test data, respectively. The vertical dashed lines indicate the epoch at which the model achieves the best macro-F1 score. We evaluated the models with the best macro-F1 score in the main text. As shown in (i), the test loss decreases only slightly due to the low predictability in this setting; higher AUC can be achieved even for high loss within this close-to-random regime. In (v) and (vi), the confusion matrices of the training and test data averaged over 6 models which achieve the best macro-F1 scores are shown. The standard deviation is also shown. Note that the total number of events are fewer than the value reported in Fig L since cells near the spatio-temporal boundaries of the data set were not used.

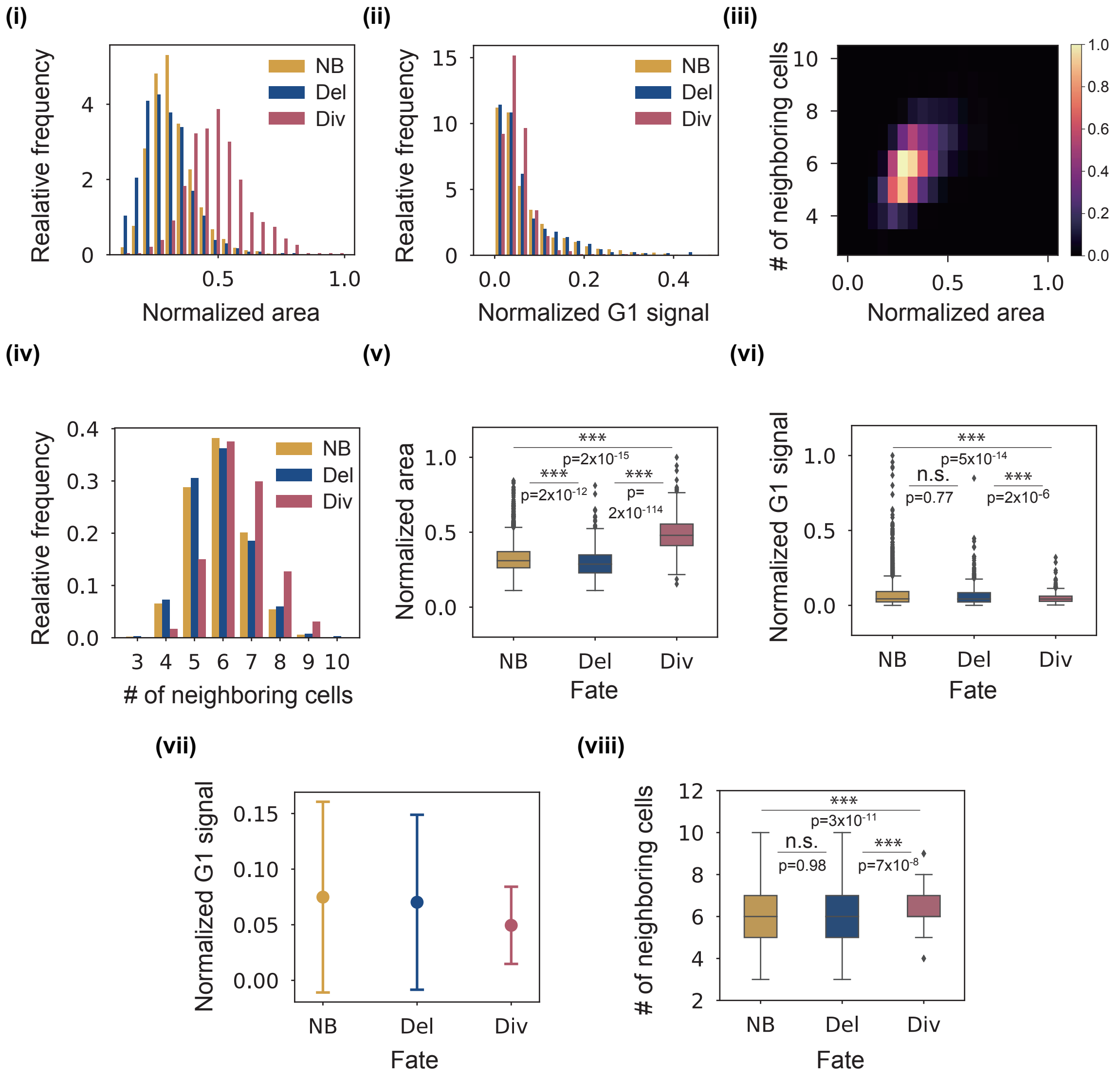

Fig C. **Statistical properties of the hind paw data.** (i,v) Histogram of relative frequency and the boxplot of the normalized area. (ii,vi,vii) Histogram of relative frequency, the boxplot, and the plot of the average of the normalized G1 signal. The error bar in (vii) is the standard deviation. (iii) The correlation matrix between the number of neighboring cells and normalized area. (iv,viii) Histogram of relative frequency, the boxplot of the number of neighboring cells. In (v,vi,viii), the significance obtained by the two-sample two-sided Kolmogorov–Smirnov test is shown ($* : p < 0.05, ** : p < 0.01, *** : p < 0.001$). In the boxplots, the box shows the quartiles of the dataset while the whiskers show the rest of the distribution. The outliers are defined by the thresholds which are obtained by multiplying the interquartile range by 1.5 and adding (reducing) it to (from) the third (first) quartile. The numbers of cells used for the analysis are 4953 (NB), 517 (Del) and 517 (Div), respectively.

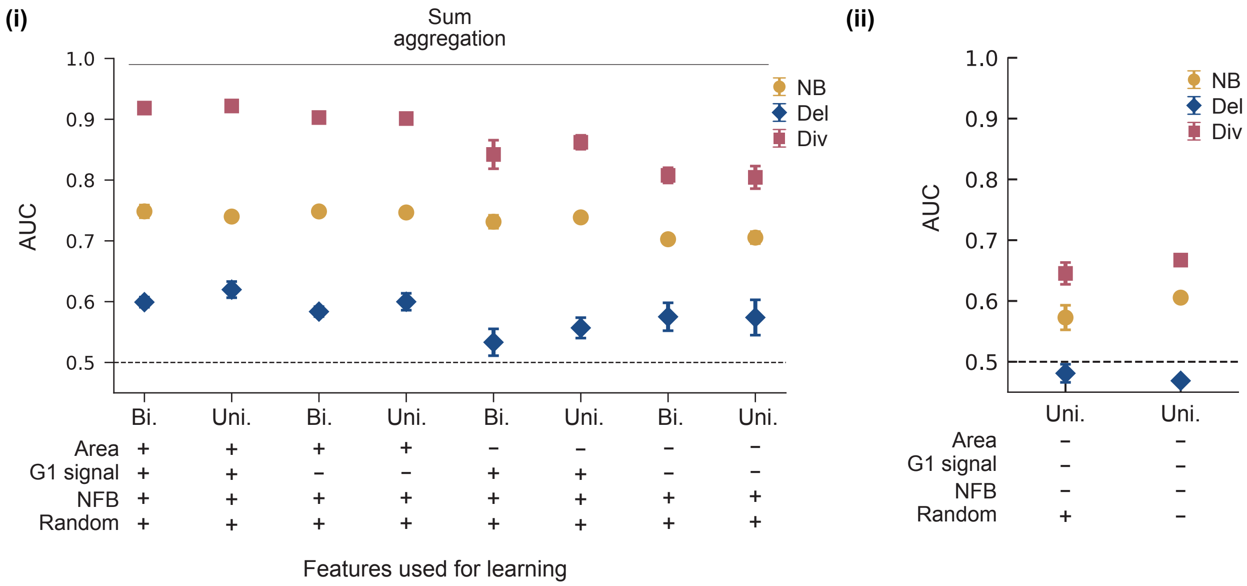

Fig D. AUC of the bidirectional spatiotemporal GNN model and the unidirectional spatiotemporal GNN model with sum aggregation. (i) The AUC of the bidirectional spatiotemporal GNN model (Bi.) and the unidirectional spatiotemporal GNN (Uni.) with sum aggregation are shown for various feature conditions obtained by averaging the AUC over six trained models. (ii) The AUC of the unidirectional GNN only with random feature and without any feature. Error bar: standard deviation.

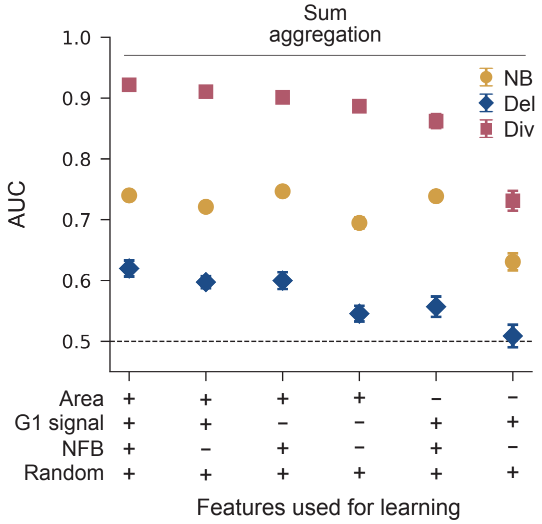

Fig E. Effect of NFB on the performance of the unidirectional spatiotemporal GNN model with sum aggregation. The AUC of the unidirectional spatiotemporal GNN for models under various feature sets obtained by averaging over six trained models. Error bar: standard deviation.

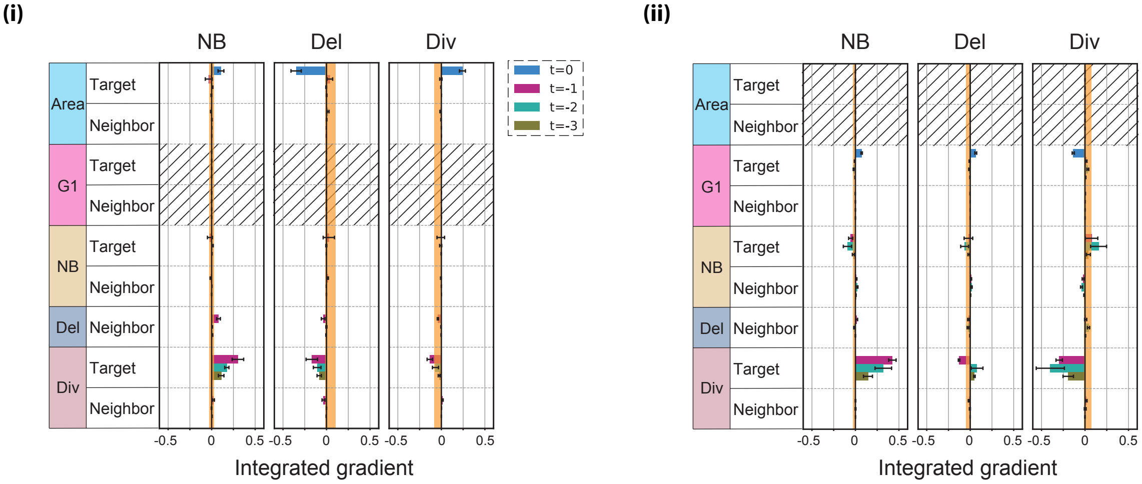

Fig F. **Attribution of unidirectional spatiotemporal GNN model with sum aggregation.** The attributions of the unidirectional spatiotemporal GNN model are shown for each feature condition: (Area, G1-phase signal, NFB, Random)= (+, −, +, +) (i) and (−, +, +, +) (ii). The IG averaged over six trained models is shown for each pooled feature. Error bar: standard error. The upper and lower values of the IGs of the random features are shown as the orange zone.

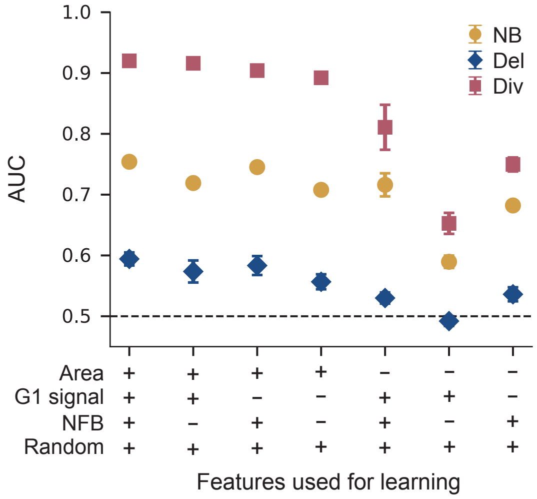

Fig G. **The performance of the unidirectional spatiotemporal GNN model without spatial MP.** The AUC of the unidirectional spatiotemporal GNN for models without spatial MP under various feature sets obtained by averaging over six trained models. We ran 5000 epochs for (Area, G1-phase signal, NFB, Random)= (−, −, +, +) because the learning was slower than the other conditions. Error bar: standard deviation.

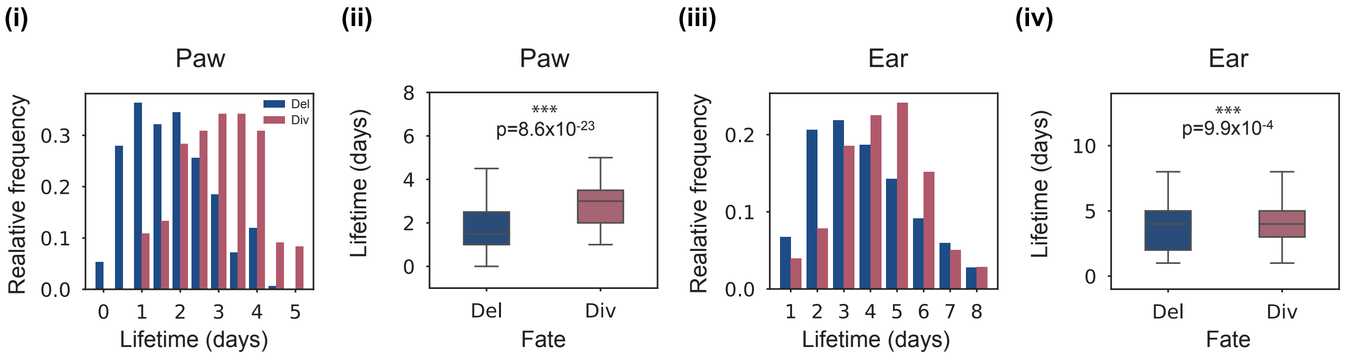

Fig H. **Lifetime distribution.** The lifetime distribution of delaminating and dividing cells as well as the box plots are presented for the (i,ii) hind paw and (iii,iv) ear. In (ii) and (iv), the significance of the two-sample two-sided Kolmogorov–Smirnov test is shown ($* : p < 0.05, ** : p < 0.01, *** : p < 0.001$). In the boxplots, the box shows the quartiles of the dataset while the whiskers show the rest of the distribution. The numbers of cells used for the analysis are 336 (Del in paw), 240 (Div in paw), 252 (Del in ear) and 178 (Div in ear), respectively.

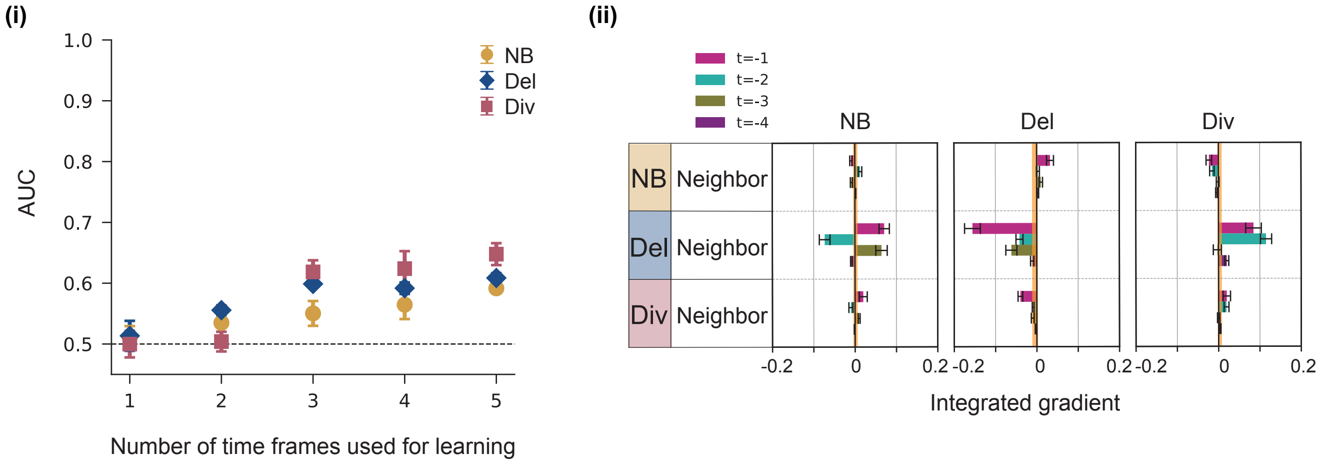

Fig I. **Dependence of the number of time frames on the performance.** The cell external model with mean aggregation was applied for the hind paw data with the feature condition: (NFB, Random)=(+, +). (i) The AUC for the different number of time frames. The AUC for each cell fate label obtained by averaging the AUC over six trained models. Error bar: standard deviation. (ii) The attribution of the five-time model. The IG averaged over six trained models is shown for each pooled feature. Error bar: standard error.

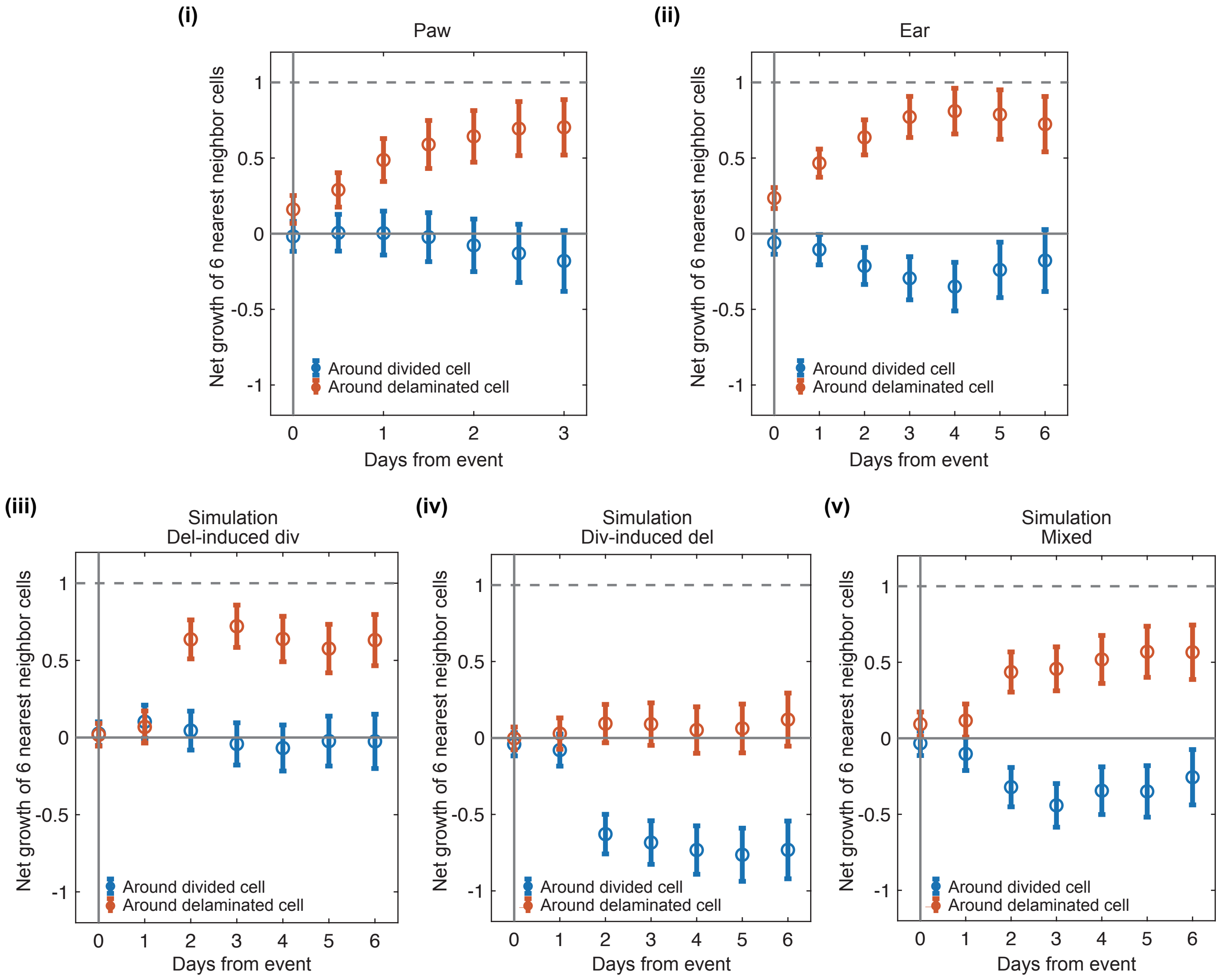

Fig J. **Neighbor fate net imbalance analysis for the epidermis data and simulations.** Net imbalance of six-nearest neighbor cells around a divided cell and a delaminated cell is plotted for the (i) hind paw and (ii) ear data, as well as for data generated by simulations in the (iii) delamination-induced division setup, (iv) division-induced delamination setup and (v) mixed setup. Error bar: standard error.

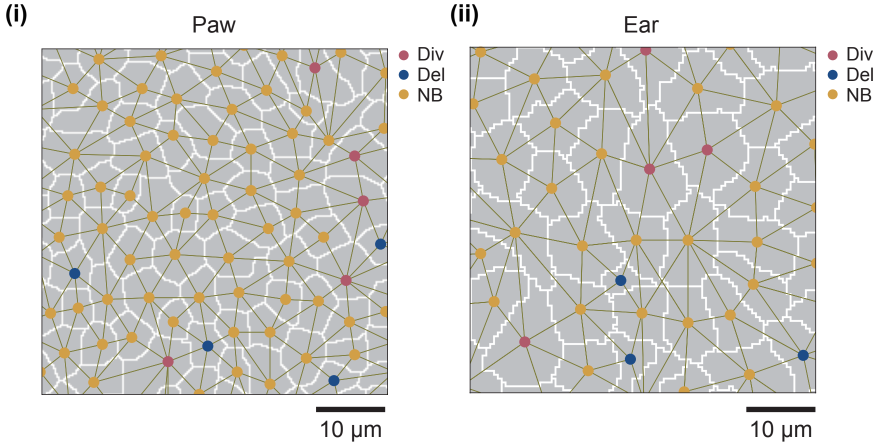

Fig K. **Segmented images of the hind paw and ear epidermis.** Segmented images are shown for the (i) hind paw and (ii) ear epidermis. Cells undergoing Div, Del, and NB are indicated by red, blue, and yellow circle markers. The cell-contact graph is also shown.

Data type	Area number	# of frames	# of NB	# of Del	# of Div	Total # of events	Training/Test
Mouse paw	1	15	2481	269	265	3015	Training
Mouse paw	2	15	2472	257	252	2981	Test
Mouse ear	1	10	1729	150	137	2016	Training
Mouse ear	2	10	1638	151	111	1900	Training
Mouse ear	3	10	2110	185	201	2496	Training
Mouse ear	4	7	1114	97	105	1316	Test
Mouse ear	5	7	1297	111	92	1500	Test
Mouse ear	6	10	3163	236	201	3600	Test
Simulation del-div	1	15	2394	261	265	2920	Training
Simulation del-div	2	15	2458	235	240	2933	Test
Simulation div-del	1	15	2501	221	237	2959	Training
Simulation div-del	2	15	2500	252	251	3003	Test
Simulation Mix	1	15	2586	262	262	3110	Training
Simulation Mix	2	15	2560	257	264	3081	Test

Fig L. Summary of the data sets.

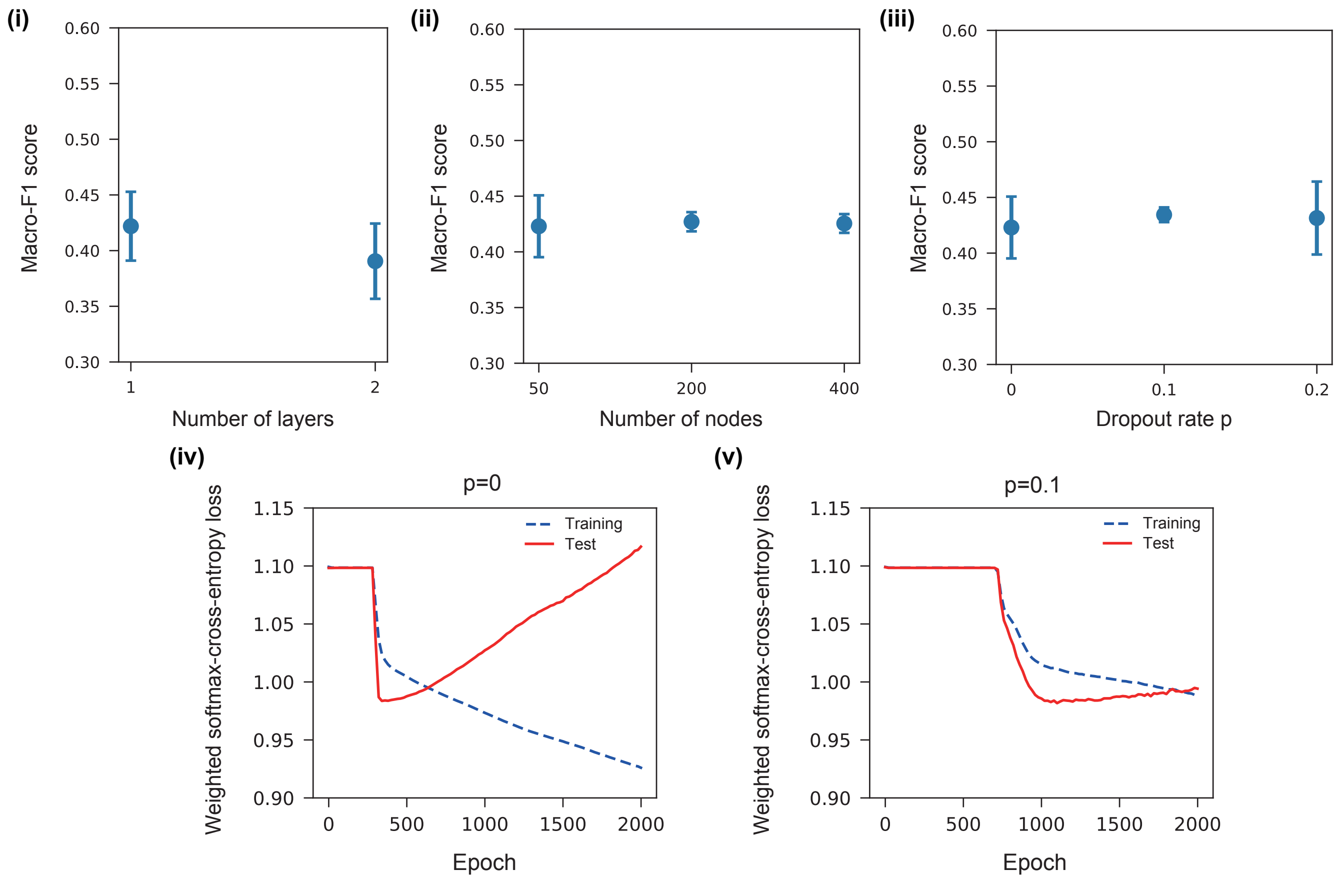

Fig M. **Dependence of the performance of the GNN models on the hyperparameters.** The cell external model of the unidirectional GNN with mean aggregation is applied to the simulation data from delamination-induced division setup with the feature condition: (NFB, Random)=(+, +). The macro-F1 score averaged over six trained models is plotted against the hyperparameters. Error bar: standard deviation. (i) The effect of N_{layer} is tested with $N_{\text{node}} = 50$ and $p = 0$. (ii) The effect of N_{node} is tested with $N_{\text{layer}} = 1$ and $p = 0$. (iii) The effect of p is tested with $N_{\text{node}} = 50$ and $N_{\text{layer}} = 1$. (iv,v) The effect of dropout on suppression of overfitting is shown. The training curves for $p = 0$ and 0.1 are shown, respectively ($N_{\text{node}} = 50$ and $N_{\text{layer}} = 1$).

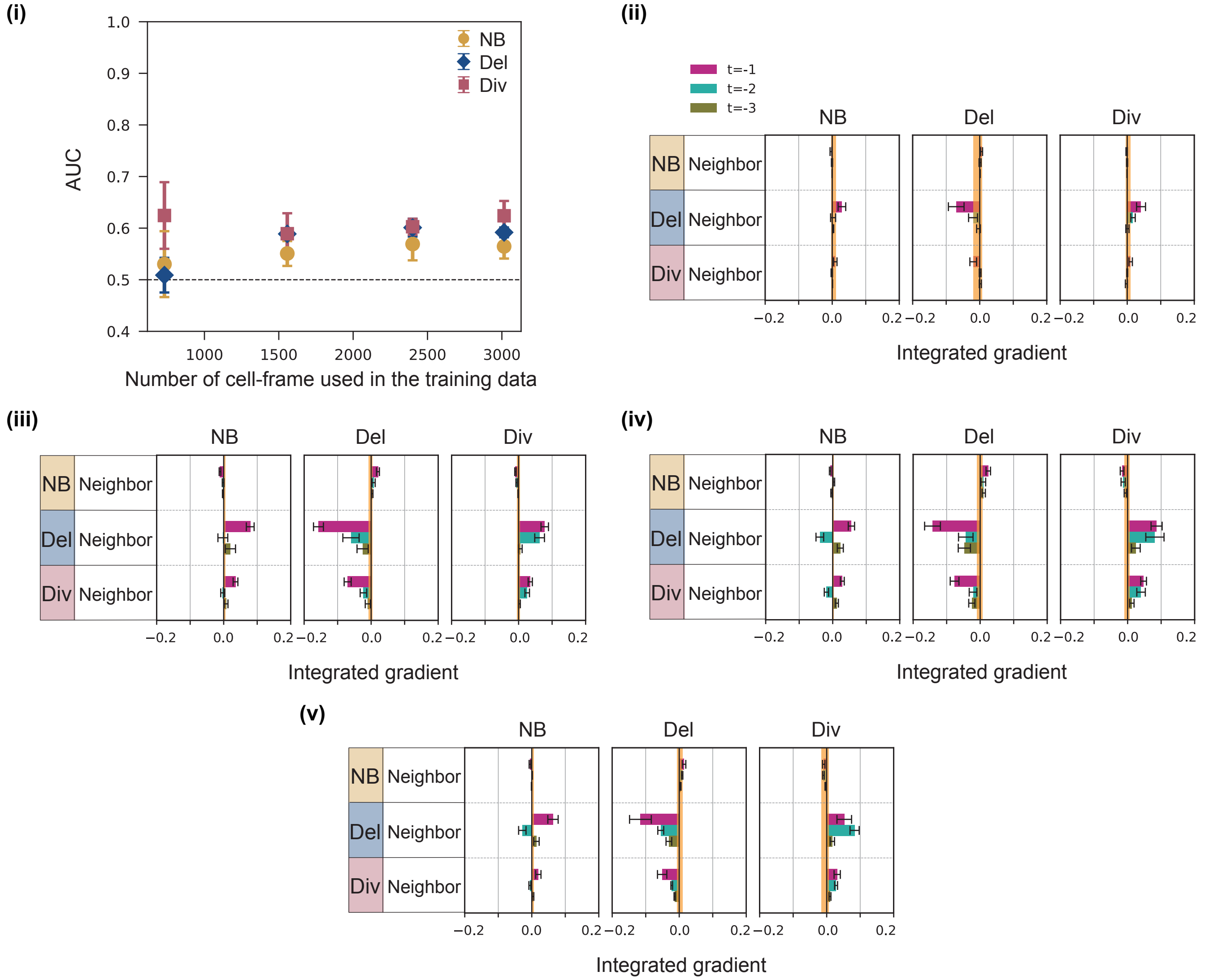

Fig N. **Data size dependency on the prediction and attribution: mouse paw data.** The cell external model with mean aggregation is applied to the mouse paw data with the feature condition: (NFB, Random)=(+, +). (i) The AUC for the different number of cell-frames in the training data obtained by averaging the AUC over six trained models. Error bar: standard deviation. (ii-v) The attribution is shown for the different numbers of cells: (ii) 731, (iii) 1557, (iv) 2400 and (v) 3015. The result in the main text is for 3015 cells in the training data. The IG averaged over six trained models is shown for each pooled feature. Error bar: standard error. The upper and lower values of the IG of the random feature are shown as the orange zone.

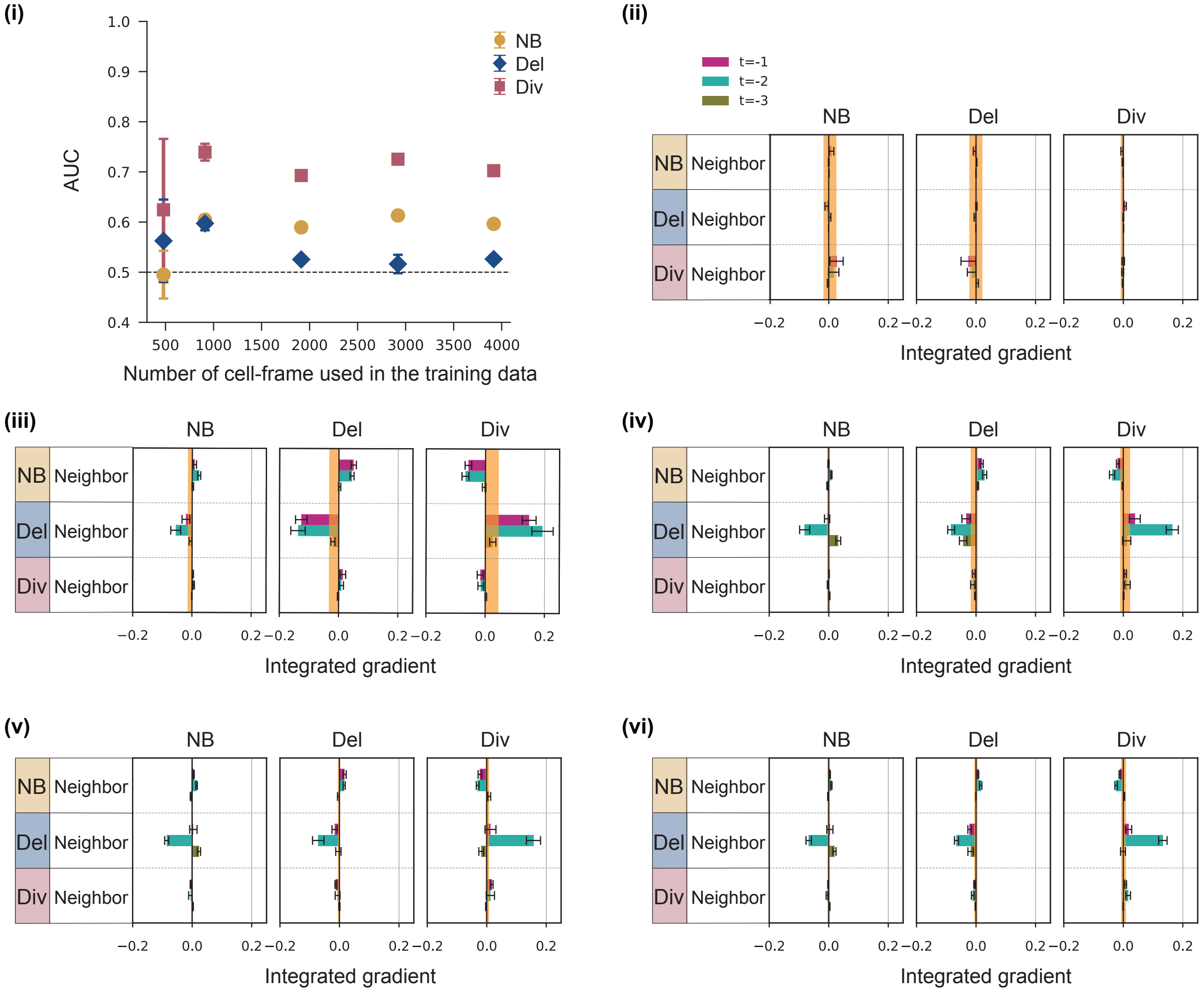

Fig O. **Data size dependency on the prediction and attribution: simulation data.** The cell external model with mean aggregation is applied to the simulation data from the delamination-induced division setup with the feature condition: (NFB, Random)=(+, +). (i) The AUC for the different number of cell-frames in the training data obtained by averaging the AUC over six trained models. Error bar: standard deviation. (ii-vi) The attribution is shown for the different numbers of cells: (ii) 479, (iii) 911, (iv) 1913, (v) 2920 and (vi) 3917. The result in the main text is for 2920 cells in the training data. The IG averaged over six trained models is shown for each pooled feature. Error bar: standard error. The upper and lower values of the IG of the random feature are shown as the orange zone.

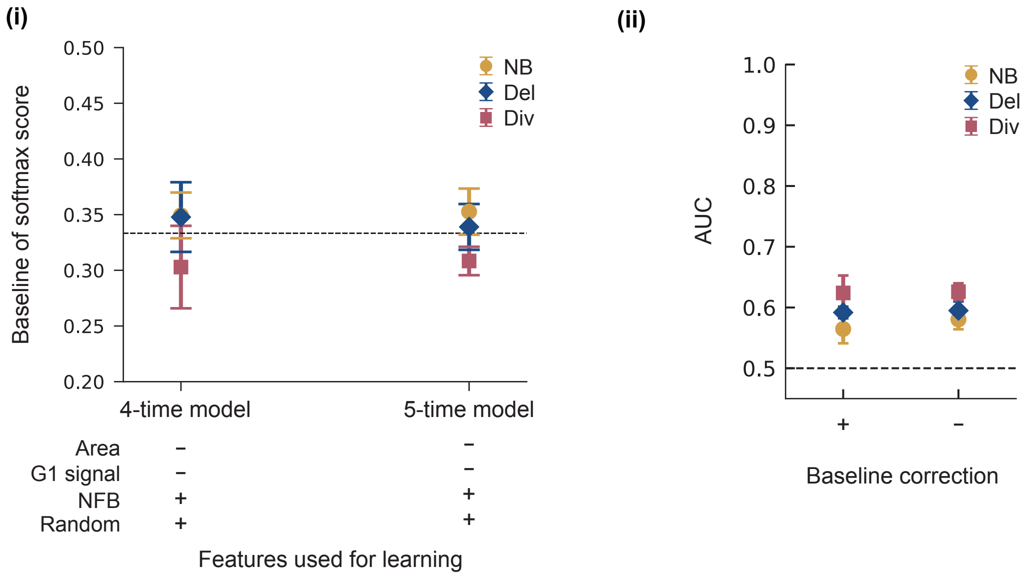

Fig P. **Baseline softmax score.** (i) The baseline softmax score, which is the softmax score for null-graphs, is shown for the four and five-time cell external model with mean aggregation for the hind paw data. The feature condition is (Area, G1 signal, NFB, Random)=(−, −, +, +). The horizontal dashed line indicates the target baseline softmax score $1/3$. (ii) The AUC is shown for the four-time cell external models with and without baseline correction.

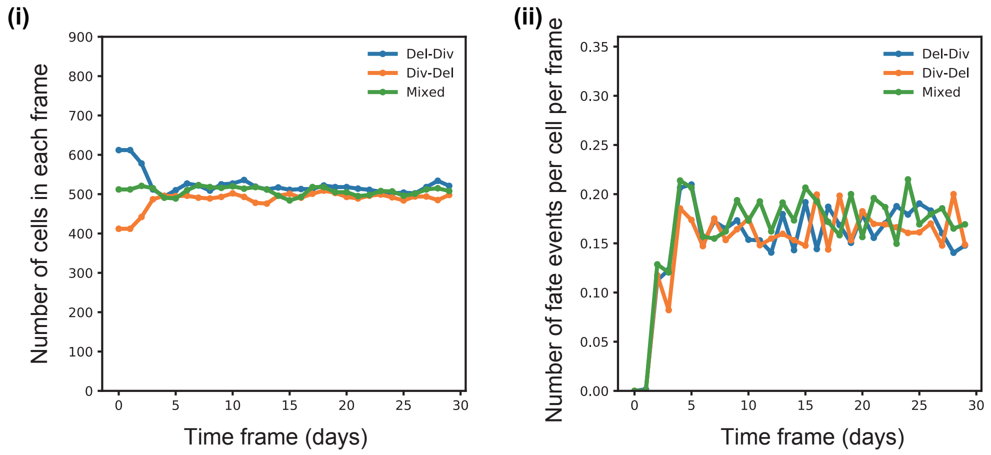

Fig Q. **Numerical simulations of the homeostatic tissue model.** (i) Time-evolution of the number of cells and (ii) the number of fate events in the simulations of the Del-induced Div model, Div-induced Del model, and the mixed model. We used the data from 15 to 30 days in these simulations for the GNN analyses.
