# Supplementary material for: Probing the rules of cell coordination in live tissues by interpretable machine learning based on graph neural networks
Source: PLoS Comput Biol. 2022 Sep 6;18(9):e1010477. doi: 10.1371/journal.pcbi.1010477 (PMC9481156; doi:10.1371/journal.pcbi.1010477)
Supplement: Attachment — Submitted filename: response.pdf [file pcbi.1010477.s003.pdf]

> Thank you very much for submitting your manuscript "Probing the rules of cell coordination in live tissues by interpretable machine learning based on graph neural networks" for consideration at PLOS Computational Biology. As with all papers reviewed by the journal, your manuscript was reviewed by members of the editorial board and by several independent reviewers. The reviewers appreciated the attention to an important topic. Based on the reviews, we are likely to accept this manuscript for publication, providing that you modify the manuscript according to the review recommendations.

> While no additional experiments are required, please note that the manuscript will only be accepted provided that the weakness points raised by Reviewer 1 and 3 (e.g. on overfitting and interpretability) are addressed by enhancing or complementing the discussion content.

We thank the editor and referees for giving us useful feedback on our manuscript. Below we answer to the referee comments one-by-one, following the summary of changes we have made in the main and supplementary text.

Summary of Changes

- We added an explanation (lines 143-147 in the main text) about our motivation to use message passing (MP) rather than convolutional/attention-based GNN to address the comments by Reviewers #1 and #3.
- We added a paragraph (lines 284-290 in the main text) emphasizing that our strategy is systematic and applicable to more complex systems to address the comment by Reviewer #3.
- We added lines 310-314 in the main text to explain that the low predictability in some cases is due to the inherent stochasticity in the cell dynamics to address the comments by Reviewers #1 and #3.
- We added lines 374-377 in the main text to complement the discussion about the interpretability of the GNN framework.
- We added Fig.S4B in S1 Appendix and the corresponding caption to show that introduction of random features does not affect the model performance to address the comment by Reviewer #1.
- We added Fig.S13D, E in S1 Appendix and the corresponding caption to show that dropout is effective to suppress overfitting to address the comment by Reviewer #1. We also added the explanation in line 508 of the main text.
- We added Fig.S16B in S1 Appendix and the corresponding caption to show that baseline correction does not affect the model performance to address the comment by Reviewer #1. We also added the explanation in lines 541-542 of the main text.
- We added references 29, 36, 37, 38, 49, and 50.
- We changed the order of reference 16 as suggested by Reviewer #2.

Reviewer's Responses to Questions

Comments to the Authors:

Please note here if the review is uploaded as an attachment.

Reviewer #1: Summary

The paper presents a framework to discover the cell coordination rules in developing tissues. Specifically, the authors consider a spatiotemporal graph constructed from the segmentation and tracking of the epidermis cells. The graph is used to train a graph neural network (GNN) on the task of cell fate prediction. Using the explanation methods from the field of explainable AI (XAI) the authors show that the trained GNN is able to learn multiple rules which control the multicellular dynamics.

The proposed combination of geometric deep learning and explanation methods is very interesting and as far as I know has not been used for analysing cell coordination in developing tissues before. Enoding the live imaging as a spatiotemporal graph and using the techniques proposed by the authors constitute a generic framework for analysis of cell interactions.

Strengths

1. The work proposes a general purpose framework for analysing interactions in spatiotemporal graphs of developing tissues.

2. It shows that even without the node features, the structure of the spatiotemporal graph can be used to accurately predict the cell fate with GNN.

3. The authors encode hypothetical cell coordination rules as additional node features and use the explanation methods to show which rules/features are most relevant for cell fate prediction.

4. The proposed method can detect previously known as well as new cell interaction rules.

5. Extensive ablation study of the method is shown in the paper and the appendix.

6. Additional results on the simulated data confirm the effectiveness of the presented method for extracting the cell interaction laws.

7. The paper is well-written and easy to follow. The source code and datasets are provided to reproduce the experiments.

We thank the reviewer for reviewing our work and for appreciating its strength and novelty.

Comments/Questions

1. The claim that the GNN is significantly better than a random guess at the cell fate classification is not strongly supported. Fig. S2 D shows the precision score is slightly above 0.8 for the NB class and between 0.1-0.2 for the Div/Del classes (test set). Given the 1:1:8 ratio between the classes (Div/Del/NB) the GNN performance is only slightly better than random.

The original claim would be more convincing if the authors show quantitative comparison with a simple baseline (e.g. shallow model trained from the node features and the aggregated features of immediate neighbours) as well as other types of GNN architectures (e.g. GCN [1], GAT [2]).

We thank the reviewer for pointing this out. According to the AUC (Fig.3B: left), we clearly find that the prediction is better than random. Indeed, the AUC score is still quite low; however, our simulation results suggest that interaction rules with stochasticity provide low AUC scores comparable to the experiments (Fig.4C). Hence, we consider that the low predictability is consistent with inherent stochasticity in the cell dynamics.

We added this explanation in the main text in the revised manuscript (lines 310-314).

We chose message passing (MP) rather than convolutional/attention-based GNN because MP has higher flexibility in representation. The generic functional form described in MP may become important to probe complex nonlinear interactions, for instance when high-dimensional data such as gene expression and morphology are used as cell features.

We added an explanation on these points in the revised manuscript (lines 143-147).

2. The learning curves in Fig. S2 A show that the model is overfitting very quickly and the checkpoint chosen based on the best F1 score is in the overfitting territory. Have the authors considered more aggressive regularization (e.g. higher dropout rate, weight decay)?

We have tried more aggressive regularization with higher dropout rates in optimizing the dropout rate in Fig.S13C. However, with the higher dropout rate (p>=0.3), the model was regularized too strongly to converge within reasonable epochs. Also, we confirmed that dropout indeed suppresses overfitting as shown in the learning curves below (D: p=0.0, E: P=0.1 of revised Fig.S13), while the model performance does not improve (Fig.S13C).

We added an explanation on the result for higher dropout rates and figures for the learning curves shown below in the revised manuscript (Fig.S13D, E).

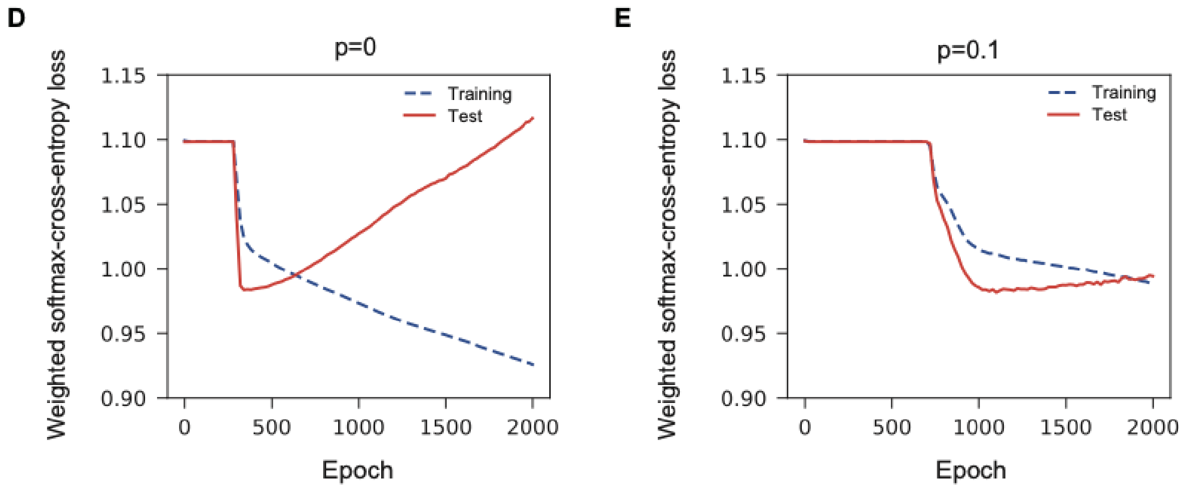

3. It would be interesting to see if the GNNs trained on the paw epidermis generalises well (without re-training) on the ear epidermis and vice versa. This could show if the rules learned by the GNN are transferable to a slightly different tissue. One could also try an external dataset such as epithelial cells of the Drosophila wing disc [3].

We thank the reviewer for the suggestion. Regarding the cross-training, we have tried training with the paw data and tested on the ear data, and vice versa, for the cell-external model as shown in the figure below. As expected, the AUC score was slightly lower compared with the case when the training and testing were conducted on the same tissue. Nevertheless, the scores were significantly higher than random, suggesting that there is some extent of transferability. This is natural since common rules are found in these tissues (Del-induced Div and Del-suppressing Del), which also show up in the attribution result as shown in the figure below.

In general, such cross-training is not guaranteed to give meaningful results, as our GNN framework is developed in order to identify the interaction rules within each tissue. For this purpose, we think it is best practice to collect enough data from one tissue type to be able to split them into training and test datasets.

We also thank the reviewer for letting us know about the dataset for Drosophila. Although we would like to consider such inhomogeneous epithelial tissues as our next target of application, we could not find an appropriate dataset at this point (the link shared in the paper seems to be outdated, and we couldn't find a public dataset in the original papers as well).

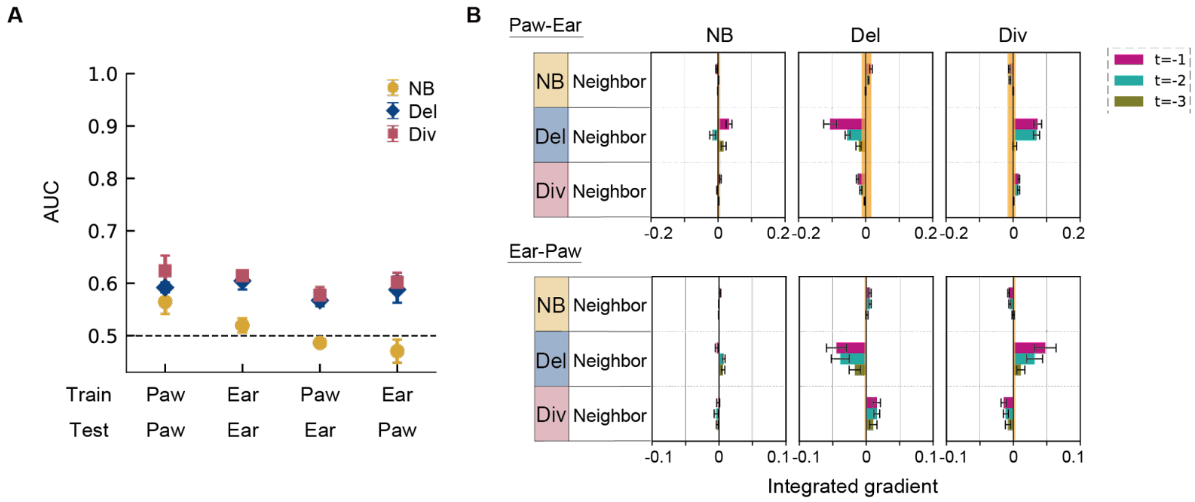

4. In order to use the integrated gradients (IG) for the attribution the authors trained the GNN with an additional MSE loss. This might negatively impact the original task of the cell fate prediction. What was the weighting factor (\alpha) for the MSE term, assuming the final loss is of the form L_{CE} + \alpha L_{MSE}? Have the authors considered simpler attribution methods which would not require training GNNs for null graphs (e.g. SmoothGrad [4], Gradient x Input [5])?

We used the weighting factor \alpha=1 for the MSE term. We did not try other methods of attribution relying on only the local differential since many of our input values are discrete and the IG method has obvious superiority. We confirmed that introducing the MSE loss does not affect the model performance as shown in the figure below. We have added this figure as Fig.S16B in the revised manuscript.

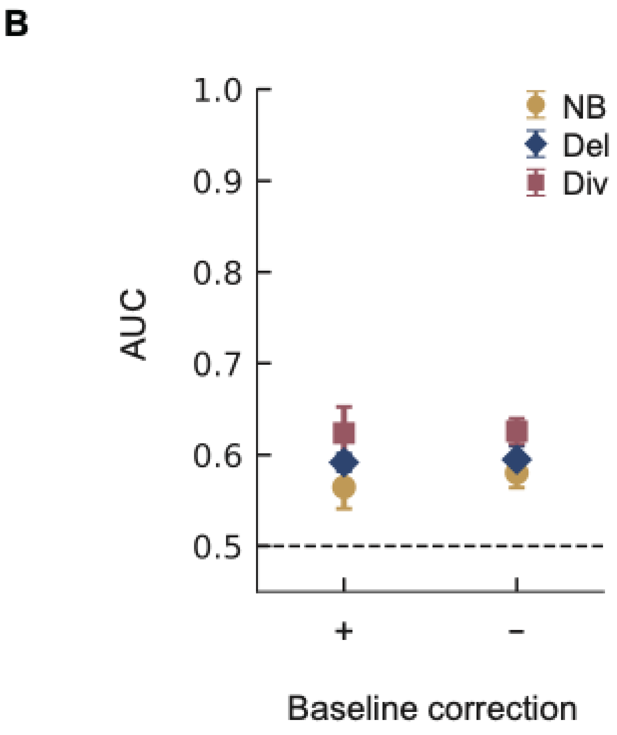

[1] T. Kipf et al. Semi-supervised classification with graph convolutional networks.

[2] P. Velickovic et al. Graph attention networks.

[3] J. Funke et al. A Benchmark for Epithelial Cell Tracking.

[4] D. Smilkov et al. SmoothGrad: Removing noise by adding noise.

[5] A. Shrikumar et al. Learning Important Features Through Propagating Activation Differences.

Reviewer #2: The authors present an approach for identifying associations between a cell's fate and features of (other) cells in its temporal and spatial vicinity. The approach is based on a spatiotemporal graph neural network (GNN) trained to predict cell fate in the final time frame, together with an attribution method (integrated gradients) that allows to identify input features relevant for the GNN's predictions.

The authors apply this framework to mouse skin cell lineage trees, and find that besides a previously reported association of cell delamination with preceding division of a spatially neighboring cell, delamination of a cell also suppresses delamination in the vicinity in subsequent timeframes, and, in densely packed tissue, cell division is associated with preceding delamination of a neighboring cell.

The proposed engineering of a framework from existing methodology (GNN architecture, IG) is well motivated, as is the training setup and the investigated model ablations. The results are convincing. In particular, while the accuracy of the GNN in terms of predicting cell fate is far from perfect / only slightly better than random in some cases (see Fig S2 F), it is still possible for IG to detect associations that are intuitively plausible, as opposed to previously reported methodology (neighboring imbalance analysis) that is less sensitive and hence only detects some but not all of the above associations.

The authors claim that their GNN architecture is novel in that it captures both spatial and temporal links. However, such kind of architecture has been used before, albeit for the cell tracking problem and not for cell fate prediction on an existing lineage tree (see e.g. https://arxiv.org/pdf/2202.04731.pdf). Nevertheless, this related line of work should be mentioned to contextualize the claimed novelty of the proposed GNN architecture.

A minor request: At some points in the manuscript references are cited not at the first mention of the respective method or finding, but somewhere later in the text (e.g., "the attribution method", and later specifically "IG"), which can be very easily fixed by just moving up citations respectively.

We thank the reviewer for reviewing our work and for appreciating the advantages in our methodology.

Following the reviewer’s advice, we added the reference as ref.29 to emphasize how GNN framework is promising in the analysis of multi-cellular dynamics. We would like to point out, however, that the original version of our manuscript was uploaded as a preprint before their work (2021.06 versus 2022.02).

We also fixed the order of ref.16 following the reviewer's request.

Reviewer #3: The Authors propose using Graph Neural Networks (GNN) as an effective and interpretable tool to analyze cell-to-cell interactions.

In particular:

-The Authors built a spatio-temporal cell adjacency graph from live-cell time-lapses and created a cell fate classification task.

-The Authors performed extensive experiments to show the strength of their GNN framework, an in-depth features analysis, and ablation studies.

-The Authors used integrated gradients (IG) as a mathematical tool to analyze features importance and interpret the GNN results.

Strengths:

Overall the manuscript showcases an interesting way of approaching biological questions, such as discovering multicellular dynamics rules. Using machine learning not only as an “automation” tool but as an instrument to understand complex interaction is a fascinating and promising (although challenging) direction.

In particular:

- the careful and detailed use of IG as a tool for dissecting the relevance of the features and time points.

- a large number of carefully crafted experiments and ablation studies.

- the manuscript reads easily, and the methods are detailed and explain all relevant implementation details well.

We thank the reviewer for reviewing our work and for appreciating its strengths.

Weaknesses:

-The experiments reported in the manuscript seem to show that the spatial MP has very little influence on the model AUC performance, i.e., it does not contribute to the GNN predictions.

This is suggested in Fig. S7 Appendix S1, where the results without spatial MP seem to be almost as good as in Fig. 2. But also in Fig. S1 Appendix S1, where it is shown that changing the Spatial interaction range does not affect the classification AUC.

Would a “standard” (for example, a multilayer perceptron) classifier with cell (no neighbor features) and temporal features (like NFB at Nt=-1 (cell parent), -2 (cell grandparent), -3..) have the same classification score? If so, what is the additional benefit of directly encoding the spatial-MP and forward-MP in a GNN compared to merely using the NFB features in a standard classifier?

As the reviewer suggests, the results for the models trained with the features of the target cells, as we show in Figs.2, S1 and S7, indicate that the features of the target cells such as cell area and NFB are the strong predictors of the cell fates, and hence elimination of the spatial MP does not significantly decrease the predictive performance. The reason for the NFB of the target cells being a strong predictor, however, comes from the fact that the division time history of the target cell provides information about its age. The cell age carries information on the cell fate, as shown in Fig.S8. The reason for the cell area being a strong predictor also has a simple explanation that cells tend to grow large before division.

The aim of our framework, on the other hand, is not to identify the strongest predictors like the cell area and NFB of the target cells, but rather to systematically identify non-trivial predictors. To this end, we demonstrated how we can first start with a model with all the features of the cells and eliminate the strong predictors one by one to arrive at the more detailed predictors such as the neighboring cell interactions.

- The seemingly little impact of the neighbor features mentioned above is not only observed in the AUC. The IG also shows minimal impact on neighbor features when the model is exposed to target features (Fig. 2), while the neighbor features relevance is high when target features are artificially “zeroed” Fig. 3 and 4. Thus, if my interpretation of the results is correct, the discovery of processes such as “Delamination-induced division” and “Lateral inhibition of delamination” (that depends strongly on the spatial neighborhood context) is not possible from the full unidirected model results but requires the model to be ad-hoc modified for this discovery.

But, in future applications, adapting the model to reveal more complex interaction processes is not straightforward and might limit the applications of the proposed framework.

Indeed, the effect of neighboring cell interactions cannot be directly probed in the full model, likely due to the existence of intermediate input features such as the change in cell area that precedes cell fate events. Nevertheless, we were able to reach more subtle effects of neighboring cell interactions by zeroing out these strong predictors. We consider that this method is clear and systematic: 1) start from a full model and identify strong predictors, 2) eliminate the strong predictors in the next model to see the next set of strong predictors, 3) continue until we have low prediction score or run out of variables.

We emphasized this procedure and its merit in the main text of the revised manuscript (lines 284-290).

-The Authors emphasize that their method is inherently more “interpretable” than alternative approaches (for example, CNN-based approaches), mainly because GNNs directly incorporate the cell spatio-temporal relationship.

But, in the manuscript, the primary source of interpretability comes from the features impact analysis rather than the graph structure. As it stands in the manuscript, there is no clear advantage of using message passing. Have the Authors thought about using attention-based GNN? An analysis of the attention pattern could lead to a more atomized understanding of which neighbor cells are important for the model to make accurate predictions.

First of all, we chose message passing (MP) rather than convolutional or attention-based GNN because MP has higher flexibility in representation. The generic functional form described in MP may become important to probe complex nonlinear interactions, for instance when high-dimensional data such as gene expression and morphology are used as cell features. We added explanations on these points in the revised manuscript (lines 143-147).

In terms of the interpretability of models, MP can also identify edge-wise attributions, using e.g. integrated gradient, similar to the attention-based GNN. Indeed, there are other methods which attribute subgraph motifs to the prediction as reviewed in [Li et al. arXiv:2203.09258, Yuan, et al. arXiv:2012.15445]. However, interpreting for instance the edge-wise attribution will require the classification of subgraph motifs, which is in general a challenging task. Instead, we here took a simpler and more convincing approach of substituting specific subgraph motifs as a feature (NFB).

We added the explanation on the future possibility of using subgraph motif attributions in the discussion section (lines 374-377).

-The Authors, between line 191 and line 197, state that the NFB features are the key ingredients (in the absence of backward-time MP) for the model performance. But in Fig. S5 Appendix S1, the second column from the left (Area, G1, NFB, rand: ++-+) suggests that even without NFB, the unidirectional model can achieve good performance using only Area and G1. In the same spirit of my first question, how powerful would a classifier be with Area and G1 as the only two features (without any MP)?

This has been presented in Fig. S7. Indeed, the Area and G1 features alone are strong predictors of fate.

Could the drastic reduction in performance you show in Fig. 2C (Area, G1, NFB, rand: ---+) compared to Fig. 1E (--) be a consequence of the random feature rather than the absence of the NFB?

We show the performance of the unidirectional model with sum aggregation in the presence (same as Fig.2C(---+)) and absence of random features in the figure below. We add this figure as Fig.S4B in the revised manuscript. The prediction score does not significantly improve without the random variable.

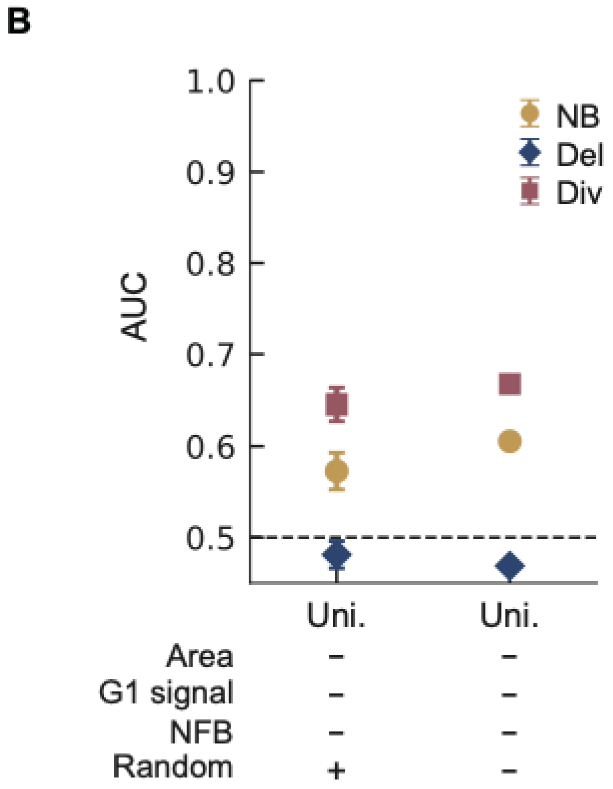

- The Authors should discuss (if possible) how and how much the error rate impacts the confidence in their biological findings. For example, is an AUC of 0.6 (such as for the Delamination detections) enough to understand the system understudy fully? In this sample case, a systematic error in the network predictions might hide significant interactions and bias the rule extracted.

What could be improved to reduce the risk of such systematic error and improve performance? For example, would a more extensive and complex set of cell features help? Or would it make results harder to interpret due to possible features cross-correlation?

We thank the reviewer for the important comment. As pointed out, in some setups, the AUC is not so high (AUC~0.6 for the cell external model applied for the epidermis data in Fig.3B and C). However, our simulation results suggest that stochastic interaction rules provide low AUC scores comparable to the experiments (Fig.4C). The reason for the low AUC in simulation can be attributed to the stochasticity in the neighbor cell choice; the neighboring coupled fate is assigned to one of the six-nearest neighbors by random choice. The low score obtained in the experimental data prediction can be explained by a similar level of stochasticity in the cell dynamics.

We do not currently know how low the AUC can be to give reliable attribution results. However, as we show in Fig.4D, we could correctly identify the rules implemented in the simulation even when the AUC is around 0.5-0.6 and the data size is about the same. This indicates that the AUC does not necessarily have to be high in order to identify the correct interaction rules.

In order to emphasize these points more, we added an explanation in the revised manuscript (line.310-314).

Recommendation:

Although I have expressed some doubts and have several questions about the proposed framework and experimental results (details in the “Weaknesses” section of my review), my overall feedback on the manuscript is positive, and I encourage the Authors to provide a revised version.

We thank the reviewer for pointing out important points and positively recommending the manuscript.

Have the authors made all data and (if applicable) computational code underlying the findings in their manuscript fully available?

The PLOS Data policy requires authors to make all data and code underlying the findings described in their manuscript fully available without restriction, with rare exception (please refer to the Data Availability Statement in the manuscript PDF file). The data and code should be provided as part of the manuscript or its supporting information, or deposited to a public repository. For example, in addition to summary statistics, the data points behind means, medians and variance measures should be available. If there are restrictions on publicly sharing data or code —e.g. participant privacy or use of data from a third party—those must be specified.

Reviewer #1: Yes

Reviewer #2: Yes

Reviewer #3: Yes

PLOS authors have the option to publish the peer review history of their article (what does this mean?). If published, this will include your full peer review and any attached files.

If you choose "no", your identity will remain anonymous but your review may still be made public.

Do you want your identity to be public for this peer review? For information about this choice, including consent withdrawal, please see our Privacy Policy.

Reviewer #1: **Yes:** Adrian Wolny

Reviewer #2: No

Reviewer #3: **Yes:** Lorenzo Cerrone
